# Supplementary material for: Adipose microenvironment promotes hypersialylation of ovarian cancer cells
Source: Front Oncol. 2024 Jul 22;14:1432333. doi: 10.3389/fonc.2024.1432333 (PMC11299042; doi:10.3389/fonc.2024.1432333)
Supplement: Supplementary file 1 [file DataSheet_1.docx]

Supporting information for:

Adipose microenvironment promotes hypersialylation of ovarian cancer cells

Alexandra Fox^1,†^, Garry D. Leonard^2,†^, Nicholas Adzibolosu^1^, Sapna Sharma^1^, Terrence Wong^1,3^, Roslyn Tedja^1,3^, Radhika Gogoi^1,3^, Robert Morris^3^, Gil Mor^1,3^, Charlie Fehl^2,3^*, Ayesha B. Alvero^1,3^*

1. C.S. Mott Center for Human Growth and Development, Department of Obstetrics and Gynecology, Wayne State University, Detroit, MI, USA; 2. Department of Chemistry, Wayne State University, Detroit, Michigan, USA; 3. Karmanos Cancer Institute, Detroit, MI, USA;

† These authors contributed equally to this work and are listed alphabetically.

Correspondence emails:
Ayesha Alvero: [ayesha.alvero@wayne.edu](mailto:ayesha.alvero@wayne.edu)

Charlie Fehl: [charlie.fehl@wayne.edu](mailto:charlie.fehl@wayne.edu)

Supplementary Material

Table of contents

| **Supplemental figures** | **Page** |
| --- | --- |
| Full Western blot images (**Supplementary Figure 1**) | 3 |
| Lectin panels (**Supplementary Figures 2-4**) | 4-5 |
| Authentication of TKO-SNA-low vs TKO-SNA-high (**Supp. Figure 5**)  Sorting of TKO-SNA-low and TKO-SNA-high subpopulations (**Supp. Figs 6-7**) | 6  7 |
| Determination of relative sialic acid levels in SNA-high vs -low cells (**Supp. Fig. 8**) | 8 |
| SNA staining of cells prior to mouse injection (**Supplementary Figure 9**) | 9 |
| Cell growth rate comparison TKO-SNA-high vs. -low (**Supplementary Figure 10**) | 9 |

# Supplementary Data

# Full Western blot images:

Full western blots used in the paper. Red box denotes region that was cropped for figures.

#
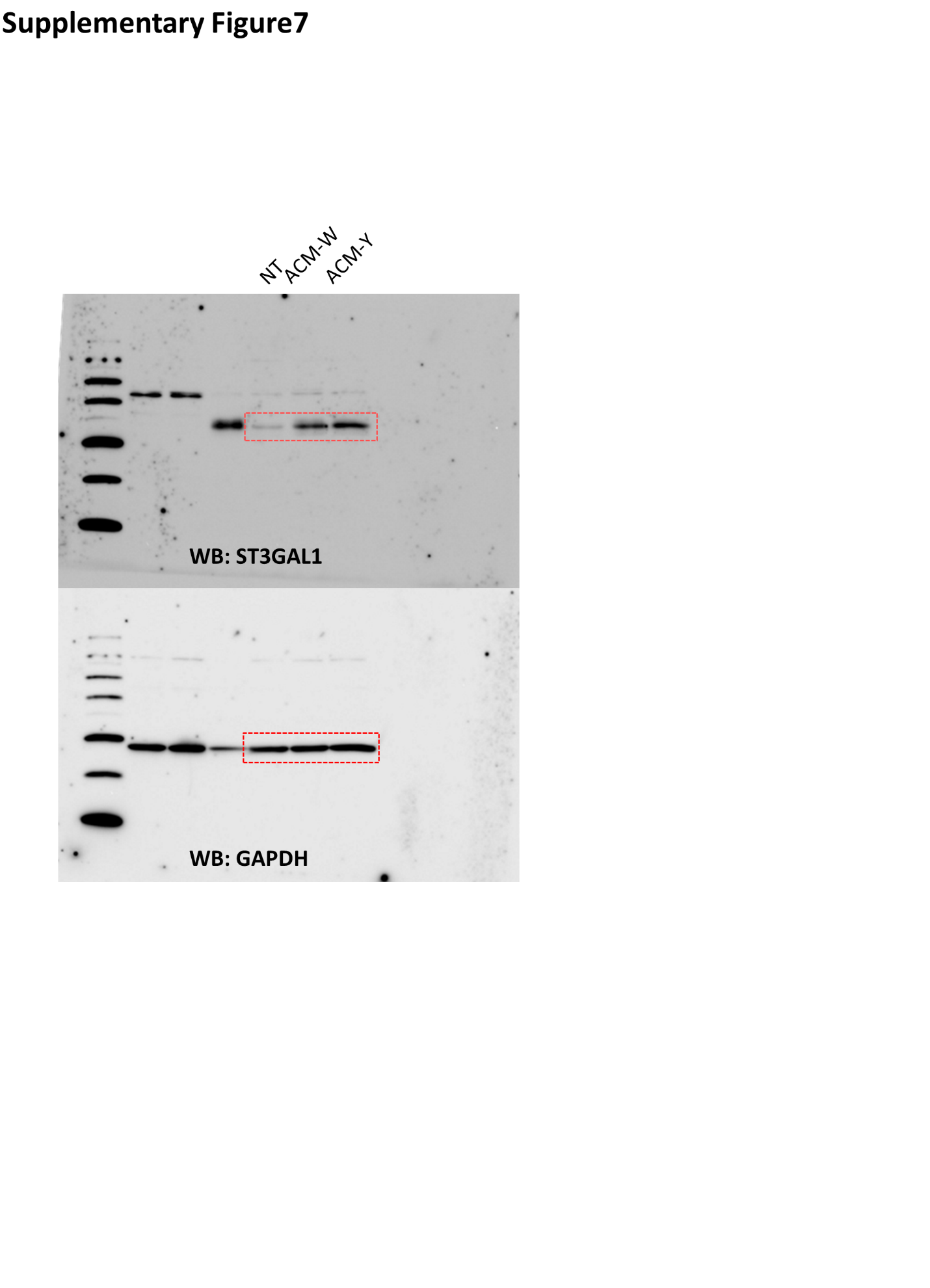


# Supplementary Figure 1: Full blots for western blot data shown in Figure 1D.

# 2 Lectin panel of ovarian cancer cell lines:

Normal cultured cell lines OCSC1-F2, R182, OVCAR3 and OVCA432, TKO or ID8p53KO were stained using SNA, Mal-I, Mal- II, and PNA. Cultured cells were detached for 4 minutes using 0.25% trypsin. The trypsin was then quenched using culture media before centrifugation at 300 g for 5 minutes. Culture media was decanted before addition of FACs buffer to resuspend cells. Cells were then passed through a 70uM filter to reduce cell clumping. Cells were then pelleted at 300 g for 5 minutes. FACs buffer was decanted and cells were then stained with 500 µL of (1:400 v/v) SNA, Mal-I, Mal-II, PNA, or PBS. Cells were stained on ice for 30 minutes. After staining, 1 mL of FACS buffer was added before pelleting and decanting liquid from pellets. Washing was repeated three times. 500 µL of FACS buffer was added to resuspend cells for flow cytometry analysis.


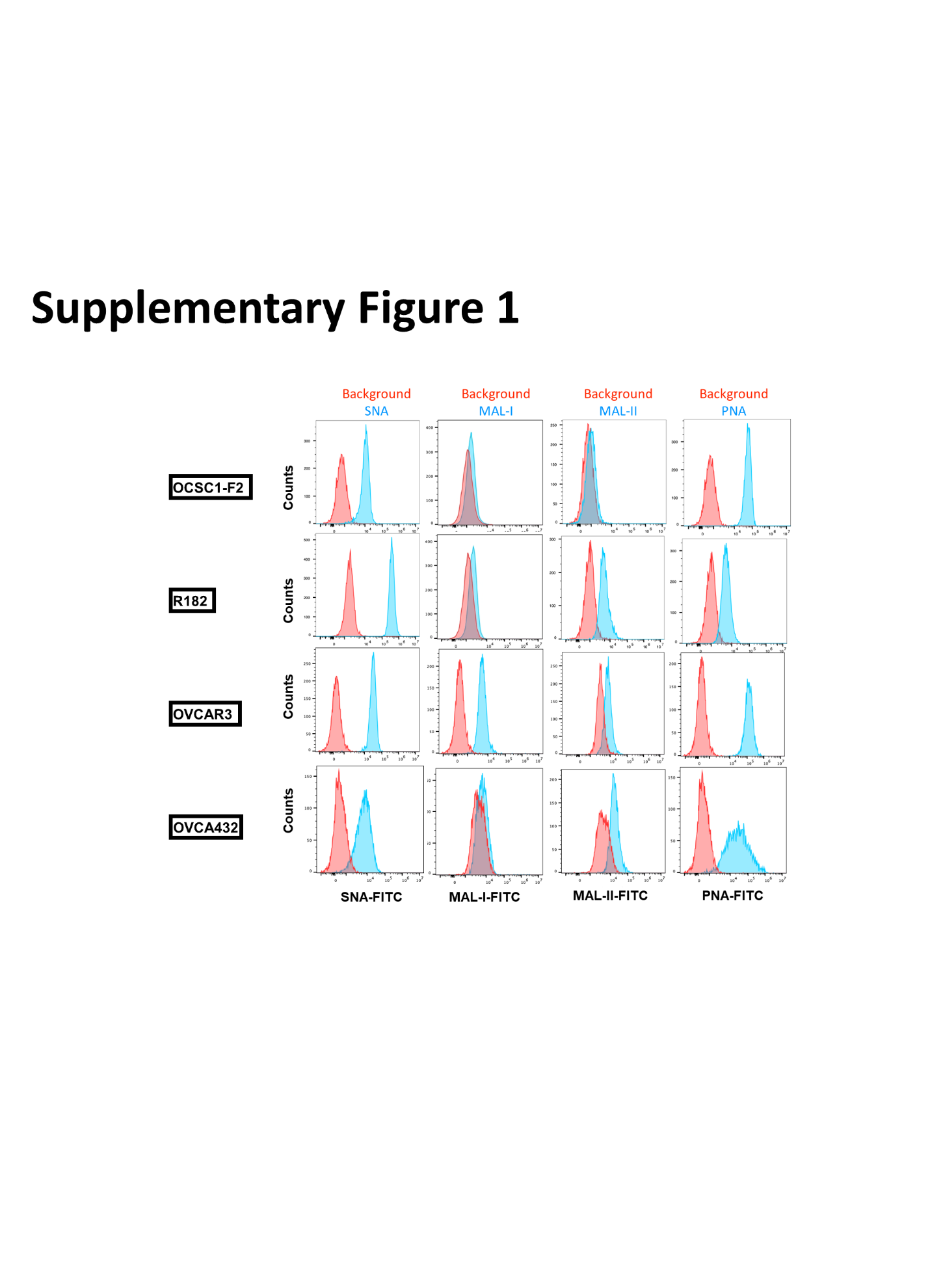


# Supplementary Figure 2: Lectin panel for human OC lines. Human OC cell lines (OCSC1-F2, R182, OVCAR3, OVCA432) were stained with SNA-FITC, Mal-I-FITC, Mal-II-FITC, or PNA-FITC.


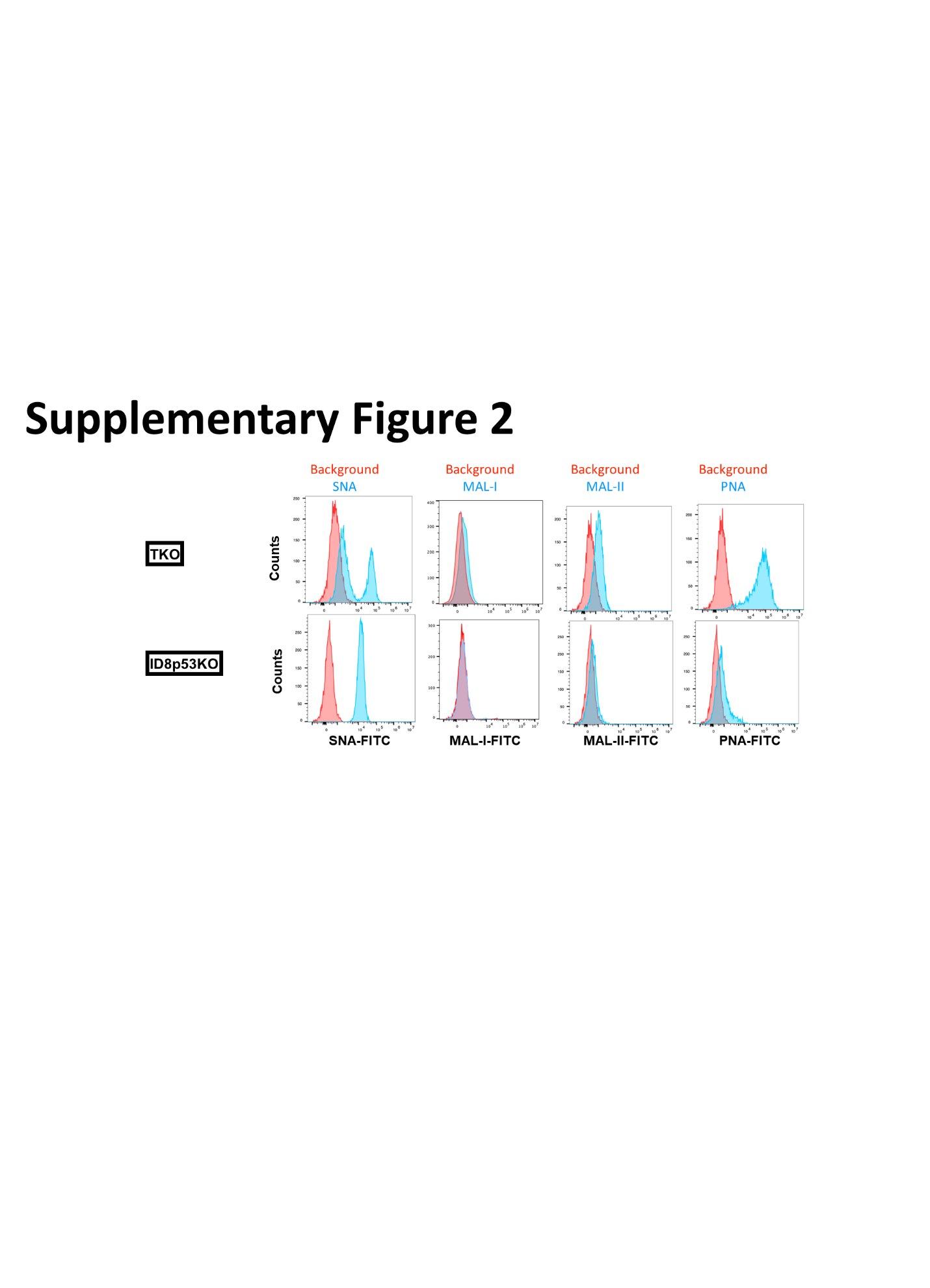


# Supplementary Figure 3: Lectin panel for mouse OC cell lines. Mouse OC cell lines (TKO and ID8*Trp53*-/-) were stained with SNA-FITC, Mal-I-FITC, Mall II-FITC, or PNA-FITC.


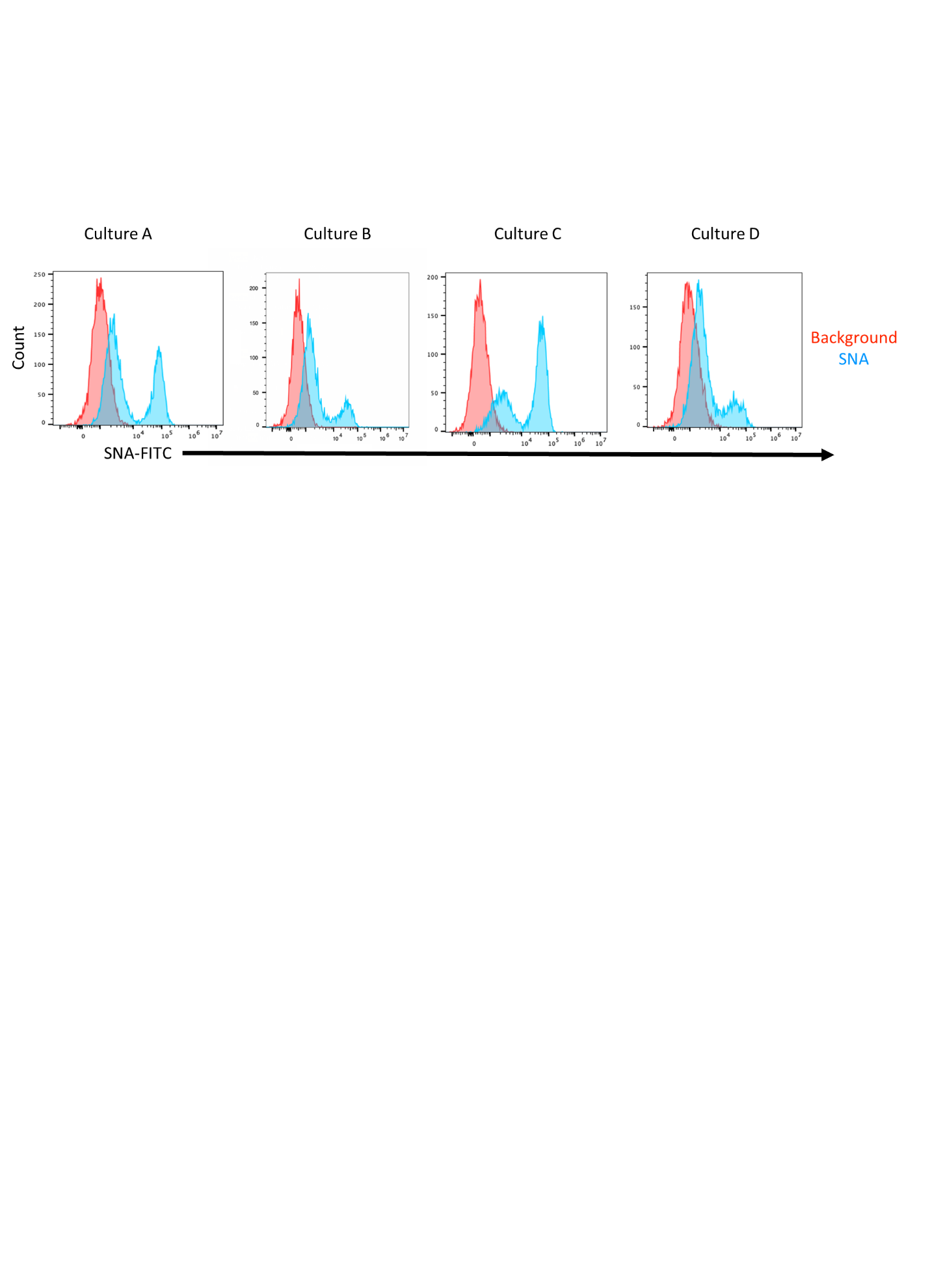


**Supplementary Figure 4:** **SNA staining for different passages of TKO mouse OC cell line.** Representative SNA staining profiles on passages of TKO ovarian cancer cells cultured between December, 2022 and March, 2023.


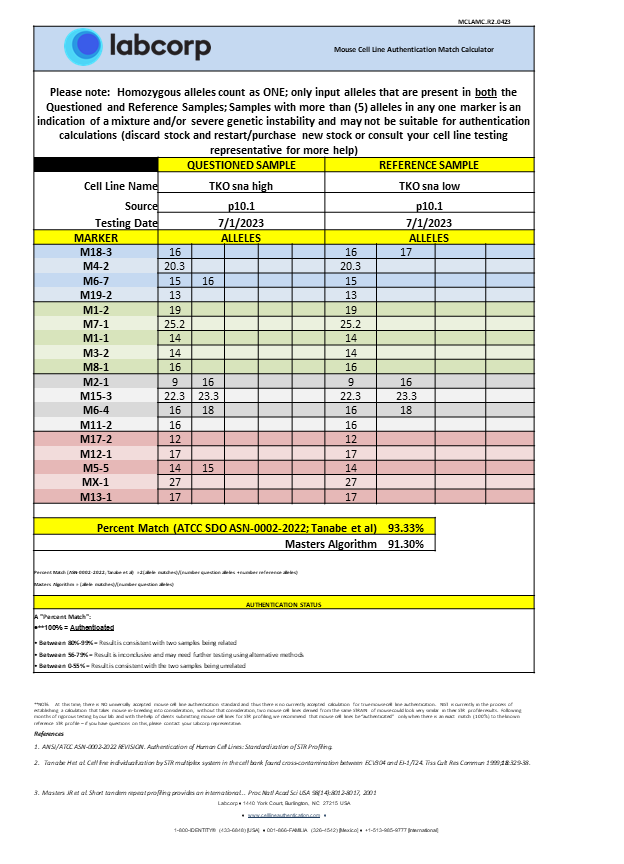


**Supplementary Figure 5:** Results of Short tandem repeat (STR)-based authentication of TKO^SNAlow^ vs TKO^SNAhigh^.


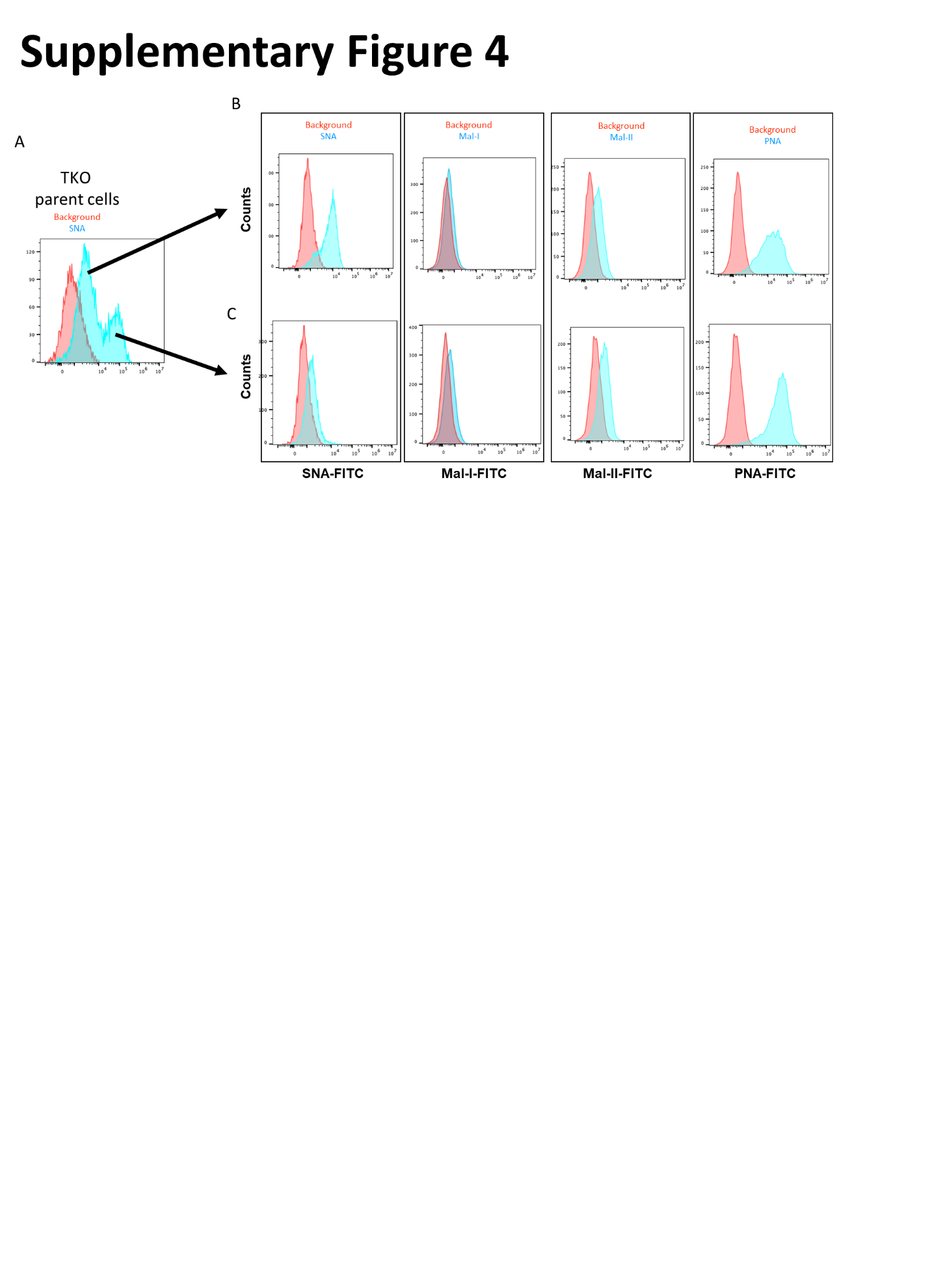


# Supplementary Figure 6: Lectin panel of TKO^SNAhigh^ and TKO^SNAlow^. Parental TKO mouse OC cells were sorted based on SNA levels and each subpopulation was stained with SNA-FITC, Mal-I-FITC, Mall II-FITC, or PNA-FITC.


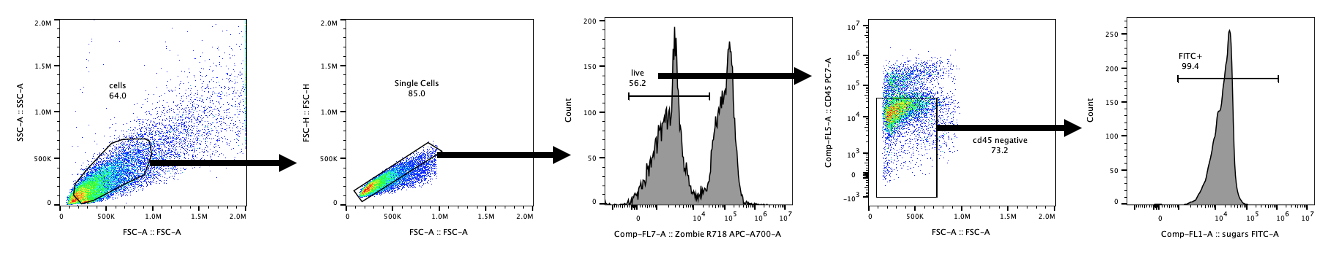


Supplementary Figure 7: Gating strategy for the analysis of lectin levels in dissociated tumors. Zombie dye was used to exclude dead cells. Only live single cells were included in the analysis.

**Determination of relative sialic acid levels in TKO high vs TKO low via neuraminidase treatment.**

To determine the relative difference in overall sialic acid between TKO high and TKO low, these ovarian cancer cell lines were subjected to neuraminidase treatment. Cells were grown in T75 culture flask in DMEM/F12 supplemented with l-Glutamine, 10% FBS, and 1% pen/strep. Cells were detached using 0.25% trypsin for 3 minutes and this was removed via media dilution and removal of the supernatant after centrifugation. Cells were counted and 1x10^6 cells were transferred to 15 mL falcon tubes. Cells were treated with 20 units of neuraminidase A (new England biolabs P0722S) in 1 mL of culture media. A separate control was also generated containing no neuraminidase. Both tubes were rotated end-over-end at 37 °C for 2 hrs. After this treatment cells were rinsed 3 times with PBS containing 10% FBS and 1% pen/strep. Cells were then subjected to PNA-FITC staining at a 1:400 (v/v) dilution for 45 minutes on ice. Cells were then rinsed 3 times with PBS before flow cytometry.


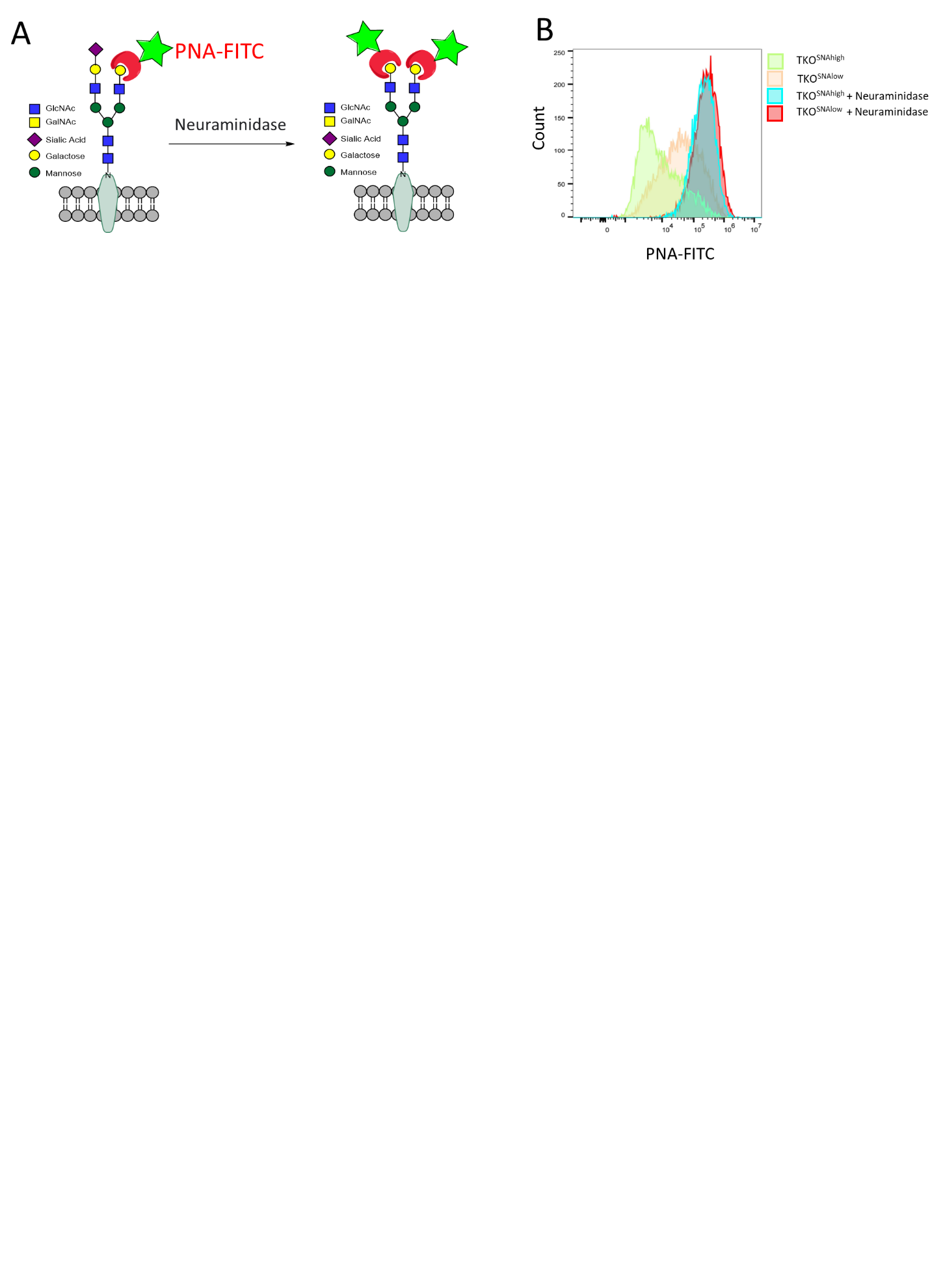
 Supplementary Figure 8: A. Model showing recognition site for PNA before and after neuraminidase treatment. B. TKO^SNAhigh^ and TKO^SNAlow^ cells were stained with PNA before after treatment with neuraminidase.


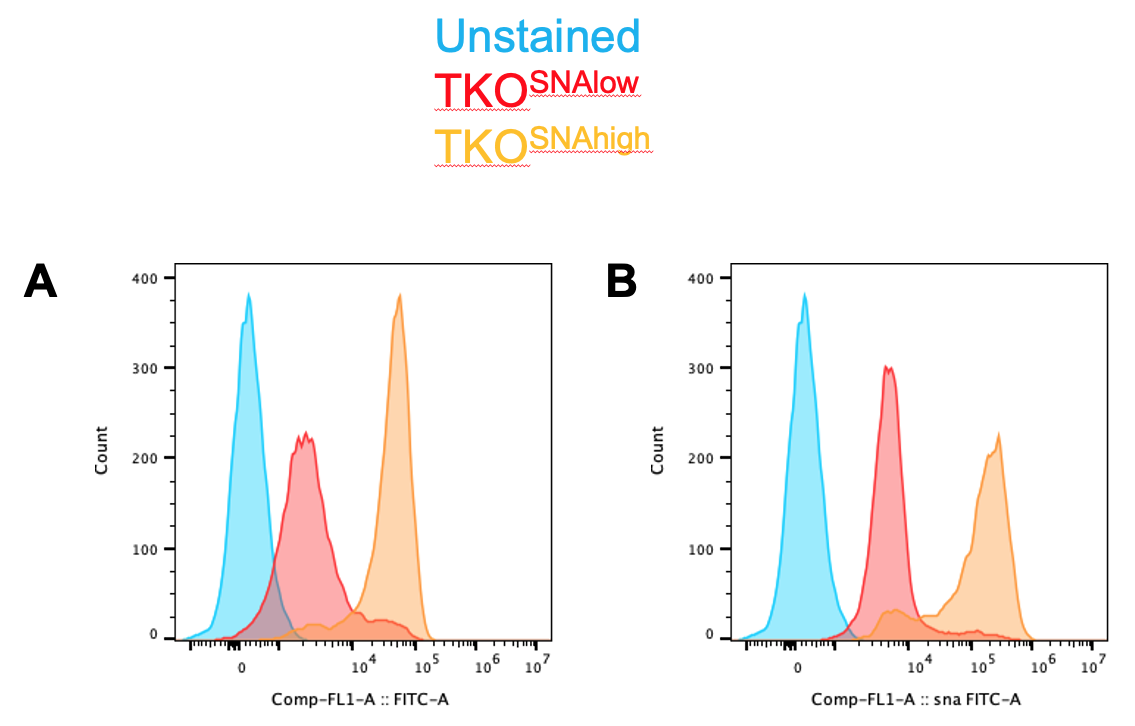


**Supplementary Figure 9:** SNA levels in TKO^SNAhigh^ and TKO^SNAlow^ cells prior to injection in C57BL6 (A) and athymic nude mice (B).


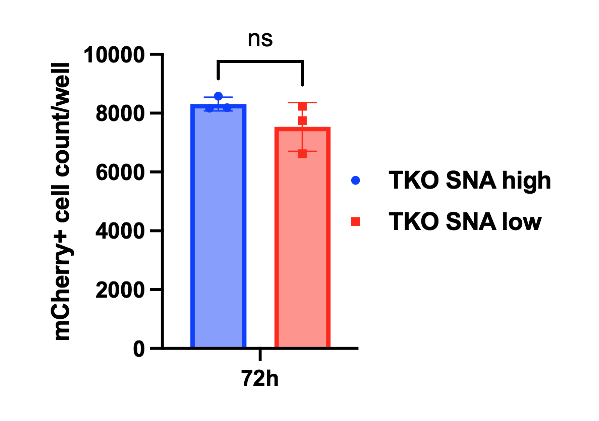


**Supplementary Figure 10:** TKO^SNAhigh^ and TKO^SNAlow^ cells have comparable cell growth in culture. Cells were seeded at 2,000 cells per well of 96-well plate. Growth was determined by quantifying the number of mCherry+ cells after 72h using Cytation 5 imager and Gen5 software. Data are presented as mean as mean ± SEM (n=3). p=0.1924 by unpaired t test.
